# Identification and Validation of Suitable Housekeeping Genes for Normalizing Quantitative Real-Time PCR Assays in Injured Peripheral Nerves

**DOI:** 10.1371/journal.pone.0105601

**Published:** 2014-08-21

**Authors:** Giovanna Gambarotta, Giulia Ronchi, Olivier Friard, Pantaleo Galletta, Isabelle Perroteau, Stefano Geuna

**Affiliations:** 1 Department of Clinical and Biological Sciences, University of Torino, Orbassano, Italy; 2 Neuroscience Institute of Turin (NIT), University of Torino, Orbassano, Italy; 3 Neuroscience Institute of the “Cavalieri Ottolenghi” Foundation (NICO), University of Torino, Orbassano, Italy; 4 Department of Life Sciences and Systems Biology, University of Torino, Torino, Italy; University of Texas at Dallas, United States of America

## Abstract

Injury to the peripheral nerve induces dramatic changes in terms of cellular composition that are reflected on RNA quality and quantity, making messenger RNA expression analysis very complex. Several commonly used housekeeping genes are regulated following peripheral nerve injury and are thus not suitable for quantitative real-time PCR normalization; moreover, the presence of pseudogenes in some of them impairs their use. To deal with this problem, we have developed a new method to identify new stable housekeeping genes based on publicly available microarray data on normal and injured nerves. Four new candidate stable genes were identified and validated by quantitative real-time PCR analysis on nerves during the different phases after nerve injury: nerve degeneration, regeneration and remyelination. The stability measure of these genes was calculated with both NormFinder and geNorm algorithms and compared with six commonly used housekeeping genes. This procedure allowed us to identify two new and highly stable genes that can be employed for normalizing injured peripheral nerve data: *ANKRD27* and *RICTOR*. Besides providing a tool for peripheral nerve research, our study also describes a simple and cheap procedure that can be used to identify suitable housekeeping genes in other tissues and organs.

## Introduction

Peripheral nerve injuries are very common casualties that may result in significant disability, thus interfering with many aspects of the life. Despite the ability of the peripheral nerve to regenerate, clinical and experimental evidences show that the regeneration is usually far from satisfactory. Therefore, the study of the mechanisms and the molecules involved in post-traumatic nerve regeneration is a key aspect to find new strategies to improve the outcome. Quantitative real-time PCR (qRT-PCR) analysis is getting more and more widely used for quantifying gene expression with high sensitivity and reproducibility. The comparison of different samples requires normalization of the rough data in relation to RNA amount and reverse transcriptase efficiency. To this end it is necessary to identify housekeeping genes (HKG) that are stably expressed, independently of the experimental conditions [1], [2], [3], [4], [5].

When the experimental conditions induce dramatic changes in the cellular composition of the tissue and/or organ under analysis, transcription of most of the genes, including commonly used HKG, could be influenced making data normalization unreliable.

To bypass the problem of the choice of the HKG, the geometric averaging of multiple HKG has been proposed as a compromise to normalize qRT-PCR data [5], based on the assumption that some HKG are up regulated while others are down regulated; however, this assumption is quite weak and the identification of stable HKG still remains the gold standard for reliable qRT-PCR analysis.

One of the organs for which identifying stable HKG is particularly troublesome is the peripheral nerve, especially when changes occurring after injury and regeneration are investigated. In fact, the cellular composition of an undamaged nerve is very poor, with limited Schwann cells, few fibroblasts and perineural cells, few resident and inactivated macrophages and some cells associated to blood vessels (endothelial cells, smooth muscle cells and perycites) [6]. After injury, the nerve segment distal to the lesion undergoes “Wallerian degeneration”, a process induced by the rapid degradation of axoplasm and axolemma which begins very early after the nerve damage [7], [8], [9]. The nerve is colonized by a large amount of phagocytic cells deriving from activation of resident macrophages as well as recruited from blood. Yet, Schwann cells, that are mitotically quiescent in the healthy nerve, dedifferentiate and start to proliferate altering dramatically their phenotype due to the loose of contact with the axons [10]. In a second phase, when new axons regrow along the aligned Schwann cell columns (bands of Büngner) [11], [12], the context changes again dramatically, due to the progressive completion of the axon and myelin debris removal and the contextual recovery of the axon-Schwann cell contact, which induces redifferentiation of Schwann cells. Finally, the process is completed by the full maturation of regenerated axons (that are remyelinated by Schwann cells) leading to the recovery of normal nerve function [13].

Since we found that commonly used HKG are not stable along the three phases of nerve regeneration (degeneration, regrowth and maturation) and, to the best of our knowledge, no previous studies were aimed at identifying reliable HKG for injured nerves, the aim of this study was to mine publicly available microarray data to identify a list of stable candidate HKG to be used in experimental peripheral nerve research. The expression stability of selected genes was then assessed by two different algorithms, geNorm [5] and NormFinder [1], leading to the identification of two new genes that showed high expression stability, irrespective of the experimental condition of the organ.

## Results and Discussion

### Commonly used housekeeping genes are regulated following peripheral nerve injury

With the aim of analyzing gene expression in peripheral nerves following injury and repair, we first tested six commonly used HKG on 12 samples corresponding to total RNA extracted 1, 7, and 28 days after median nerve crush lesion, and from control uninjured nerves, in biological triplicate. We observed that the amount of total RNA extracted from an healthy nerve is much less than the RNA extracted from an injured nerve in which the cells, involved in nerve degeneration and regeneration, have an intense transcriptional activity. Transcriptional activity gets back to a normal level at the end of the regeneration process, when the amount of total RNA is again low. These relevant changes in the total amount of extracted RNA are one of the factors contributing to the difficulties in identifying stably expressed genes during nerve degeneration and regeneration.

**Figure 1:**
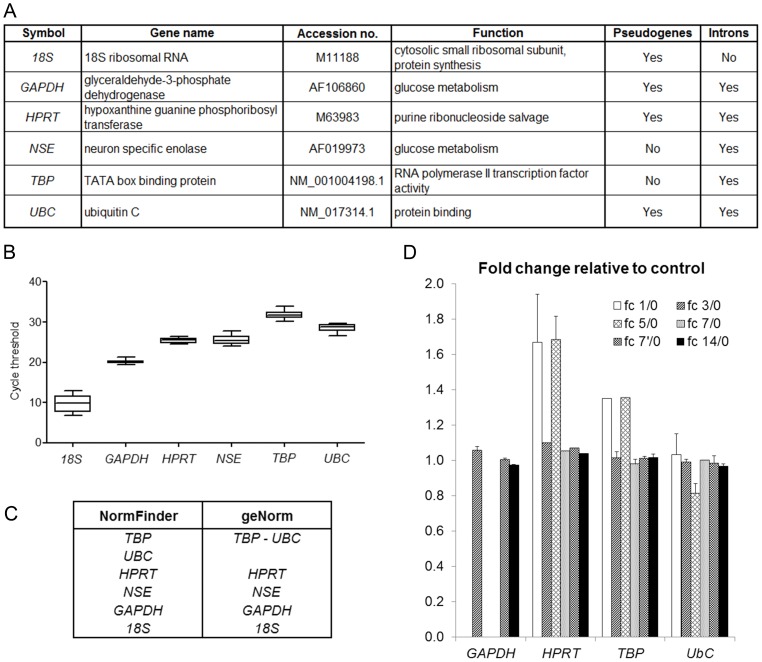
Stability analysis of six commonly used housekeeping genes. *Panel A* - List of the six analyzed genes. Primers were designed on the sequence corresponding to the accession number. The presence of pseudogenes and introns was determined by BLAST search on the rat genome (April 2014). *Panel B* - Box-plot graph of cycle threshold values of the six HKG in 12 samples. Graphs are represented as medians (lines), 25th percentile to the 75th percentile (boxes) and ranges (whiskers) for all samples. *Panel C* - Ranking of the genes according to their expression stability (increasing from bottom to top) calculated on 12 samples. *TBP* and *UBC* are the more stable genes according to both NormFinder and geNorm algorithms. *Panel D* - Fold change expression of commonly used HKG in injured nerves relative to control uninjured nerve, according to three published microarray data. The ratios were obtained by comparing the normalized fluorescence intensity at different time points after injury, with the fluorescence intensity of the control nerve. Average fold change (fc) relative to control uninjured nerve at different time points was analyzed: 1 day (fc 1/0) and 5 days (fc 5/0) [14], 7 days (fc 7/0) [15], 3 (fc 3/0), 7 (fc 7′/0) and 14 (fc 14/0) days [16]. *18S* and *NSE* data were not found in the three analyzed microarrays, whereas *GAPDH* was found in only one microarray [16].

RNA was retro-transcripted and cDNA was analyzed for the expression of the following six commonly used HKG (Fig. 1A): 18S ribosomal RNA (*18S*), Glyceraldehyde-3-phosphate dehydrogenase (*GAPDH*), Hypoxanthine guanine phosphoribosyl-transferase (*HPRT*), Neuron Specific Enolase (*NSE*), TATA box binding protein (*TBP*), Ubiquitin C (*UBC*) [5]. Threshold cycles (CT) of each HKG for each sample were obtained and plotted in a box plot graph (Fig 1B). The six reference genes were differently expressed, ranging from 9.85 (*18S*, the highest expression) to 31.71 (*TBP*, the lowest expression).

Moreover, some HKG are characterized by the presence of multiple pseudogenes in the genome (i.e. *GAPDH, HPRT, UBC*) or by the absence of introns (i.e. *18S*) (Fig. 1A). Performing a DNAse reaction to eliminate genomic DNA traces or a reaction without the reverse transcriptase enzyme to quantify genomic DNA contamination is not recommended when RNA amount is low (like in peripheral nerve tissue). Therefore, in order to be sure that genomic DNA traces are not amplified during PCR, it is important that HKG do not present pseudogenes and contain more than one exon, in order to design primers on different exons, separated by an intron, ideally longer than 1000 bp.

The CT of the six HKG were analyzed by NormFinder and geNorm and, with both algorithms, *TBP* and *UBC* resulted to be the two more stable genes (Fig. 1C). However, both genes have relevant shortcomings, namely *UBC* presents pseudogenes and *TBP* expression level is really low in peripheral nerve tissue, thus providing further support to the need to identify novel and reliable stable HKG for peripheral nerve analysis.

### Identification of new candidate housekeeping genes by microarray analysis

To identify new stable HKG, we took advantage of publicly available microarray data obtained with RNA extracted from mouse injured sciatic nerve samples. Microarray data from the following three independent databases were found and analyzed:

1. control nerves compared with injured nerves 1 and 5 days after sciatic nerve cut [14].
2. control nerves compared with injured nerves 7 days after sciatic nerve cut [15].
3. control nerves compared with injured nerves 1, 3, 7 and 14 days after sciatic nerve microcrush [16].

To identify not regulated genes, our attention was initially focused on microarrays performed on RNA extracted 1 and 5 days after sciatic nerve cut [14], because the strongest environmental variations occur at early stages after injury. Fold changes (fc) corresponding to the ratio between fluorescence at 1 day after lesion and fluorescence of control samples (fc_1/0_) were calculated. Microarray chip contained 25697 probes; those with a fc_1/0_ between 0.99 and 1.01 were 1840.

Then, fc corresponding to the ratio between fluorescence at 5 days after lesion and fluorescence of control samples (fc_5/0_) were calculated; among genes with a fc_1/0_ between 0.99 and 1.01, those with a fc_5/0_ between 0.99 and 1.01 were 192. Among them, those with a fc_5/1_ (ratio between fluorescence at 5 days and fluorescence 1 day after lesion) between 0.99 and 1.01 were 132.

To obtain sufficiently expressed genes, all genes with a fluorescence signal <100 were discarded (66 genes).

In the microarray chip, each gene is represented by one or more probes. To reduce the number of candidate genes and to increase the probability of finding stable genes, we selected only those represented by more than one probe, reducing the list to 39 genes.

Then, to confirm candidate gene stability, microarray data obtained with RNA extracted 7 days after sciatic nerve cut [15] were analyzed and the ratio between fluorescence at 7 days after lesion and fluorescence of control samples (fc_7/0_) was calculated.

All probes for each selected gene were identified and the average and standard deviation of fc_1/0_, fc_5/0_, fc_5/1_ and fc_7/0_ were calculated.

**Figure 2:**
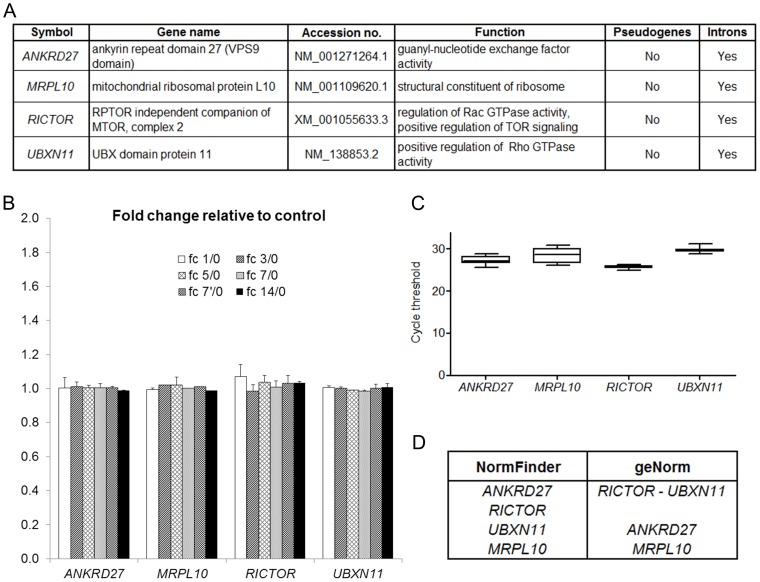
Stability analysis of four new candidate genes. *Panel A* - List of the four genes identified by microarray analysis. Primers were designed on the sequence corresponding to the accession number. The presence of pseudogenes and introns was determined by BLAST search on the rat genome (April 2014). *Panel B* - Fold change expression of the four candidate genes in injured nerves relative to control uninjured nerve, according to three published microarray data. The ratios were obtained by comparing the normalized fluorescence intensity at different time points after injury, with the fluorescence intensity of the control nerve. Average fold change (fc) relative to control uninjured nerve at different time points was analyzed: 1 (fc 1/0) and 5 days (fc 5/0) [14], 7 days (fc 7/0) [15], 3 (fc 3/0), 7 (fc 7′/0) and 14 (fc 14/0) days [16]. *Panel C* - Box-plot graph of cycle threshold values of the four HKG in 12 samples. Graphs are represented as medians (lines), 25th percentile to the 75th percentile (boxes) and ranges (whiskers) for all sample. *Panel D* - Ranking of the genes according to their expression stability (increasing from bottom to top) calculated on 12 samples. *ANKRD27* and *RICTOR* are the more stable genes according to NormFinder, *RICTOR* and *UBXN11* according to geNorm.

Only four genes with fc_1/0_, fc_5/0_, fc_5/1_ and fc_7/0_ average between 0.98 and 1.02, standard deviation ≤0.05 and absence of pseudogenes were identified: *ANKRD27* (Ankyrin repeat domain 27), *MRPL10* (mitochondrial ribosomal protein L10), *RICTOR* (RPTOR Independent Companion Of MTOR, Complex 2), and *UBXN11* (UBX domain protein 11) (Fig. 2A).

To further confirm the stability of these four genes, microarray data obtained 1, 3, 7 and 14 days after sciatic nerve microcrush were analyzed [16] and the corresponding fc was calculated. This analysis demonstrated that the expression of these four genes is stable also in this experimental model analyzed at different time points.

To conclude, we can affirm that these four genes were stably expressed according to 15 different microarrays (some performed in technical replicate) analyzed by three independent groups, using different nerve injury models (Fig. 2B).

Using the same microarray data, average Fc at different time points for commonly used HKG, *GAPDH*, *HPRT*, *TBP*, *UBC* (*18S* and *NSE* were not found) was also calculated and we confirmed that the expression of these genes is regulated following nerve injury (Fig. 1D).

For each of the four new candidate genes (*ANKRD27*, *MRPL10*, *RICTOR*, *UBXN11*) primers were designed on different exons, not affected by known alternative splicing, separated by a large intron (Table 1). A preliminary qRT-PCR was performed on 12 samples corresponding to RNA extracted 1, 7 and 28 days following nerve crush and control uninjured nerves, in biological triplicate. Raw CT values were plotted in a box plot graph (Fig. 2C); CT values ranged from 25.65 (*RICTOR*) to 29.65 (*UBXN11*). Finally, the CT of the four new HKG were analyzed by both geNorm and NormFinder and results showed that the two more stable genes were either *ANKRD27* and *RICTOR* (NormFinder algorithm) or *RICTOR* and *UBXN11* (geNorm algorithm) (Fig. 2D).

**Table 1:** Primer sequences of the 10 genes analyzed in this study: 6 commonly used HKG and 4 new candidate HKG.

Symbol	forward primer 5′-3′	reverse primer 5′-3′	amplicon size	intron spanning
*18S*	CGATGGTAGTCGCCGTGCC	CCGTTTCTCAGGCTCCCTCTCC	84	No
*GAPDH*	CCACCAACTGCTTAGCCCCC	GCAGTGATGGCATGGACTGTGG	91	Yes
*HPRT*	GCCCCAAAATGGTTAAGGTTGCAAG	ATCCAACAAAGTCTGGCCTGTATCC	81	Yes
*NSE*	GCGGCTTTGCCCCCAATATCC	ATGGCTTCCTTCACCAGCTCC	61	Yes
*TBP*	TAAGGCTGGAAGGCCTTGTG	TCCAGGAAATAATTCTGGCTCATAG	68	Yes
*UBC*	TCGTACCTTTCTCACCACAGTATCTAG	GAAAACTAAGACACCTCCCCATCA	82	Yes
*ANKRD27*	CCCAGGATCCGAGAGGTGCTGTC	CAGAGCCATATGGACTTCAGGGGG	95	Yes
*MRPL10*	CTCCTCCCAAGCCCCCCAAG	CAGACAGCTATCATTCGGTTGTCCC	97	Yes
*RICTOR*	GAGGTGGAGAGGACACAAGCCC	GGCCACAGAACTCGGAAACAAGG	81	Yes
*UBXN11*	GCGAGACTGGATGAAGGCCAAG	CCCTCCACCACCAGCTCACTC	120	Yes

### Validation of the stability of candidate housekeeping genes

**Figure 3:**
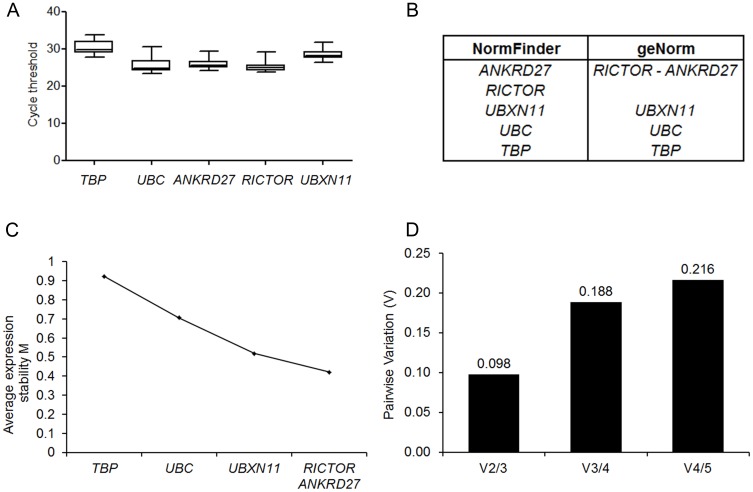
Identification of the most stable genes in the injured peripheral nerve. *Panel A* - Box-plot graph of cycle threshold values of five HKG (2 commonly used and 3 new candidates) analyzed in 40 samples. Graphs are represented as medians (lines), 25th percentile to the 75th percentile (boxes) and ranges (whiskers) for all sample. *Panel B* - Ranking of the genes according to their expression stability (increasing from bottom to top) calculated on 40 samples. *ANKRD27* and *RICTOR* are the more stable genes according to both NormFinder and geNorm programs. *Panel C* - Average expression stability value (M) of genes remaining during stepwise exclusion of the least stable gene, according to geNorm: the lower the M, the more stable the gene. *Panel D* - Pairwise variation (V) analysis to determine the optimal number of control genes required for accurate normalization; according to geNorm [5] a cutoff of 0.15 for V was used and two genes are sufficient.

The expression of *TBP* and *UBC* (the two most stable genes among commonly used HKG) and of *ANKRD27*, *RICTOR* and *UBXN11* (the three most stable genes among new candidate HKG) was analyzed on a series of 40 experimental nerve samples obtained at different time points (see materials and methods) following nerve crush or complete nerve cut and end-to-end repair. Figure 3A represents the row CT of the five housekeeping genes, which range from 25.02 (*RICTOR*, highest expression) to 30.31 (*TBP*, lowest expression). CT were then analyzed by both NormFinder and geNorm and the two more stable genes were found to be *ANKRD27* and *RICTOR* (Fig. 3B,C) with both algorithms. We finally determined the pairwise variation (V) (Fig. 3D) to define the optimal number of reference genes required for accurate data normalization. According to geNorm [5], a V value of below 0.15 has been suggested as the optimal score. In our experimental model, two genes are sufficient for normalization (V2/3 = 0.098). Indeed, when a third gene is included, the V value is 0.188, meaning that the addition of the third HKG is unnecessary to normalize gene expression.

## Conclusions

*ANKRD27* and *RICTOR* were identified as highly stable genes along all phases of peripheral nerve degeneration and regeneration and are thus suitable HKG for normalization of gene expression data.

The identification of new HKG provides a useful tool for the study of the peripheral nerve regeneration, which has become a hot topic in regenerative medicine and tissue engineering. Indeed, the use of stably HKG is necessary to normalize data that are going to be shared with the scientific community, because data normalized with an incorrect HKG can lead to a wrong interpretation of the results.

Moreover, this study shows that publicly available microarray data can be successfully used in order to identify stably expressed genes. The method that we have developed can thus be widely applied to other tissues and organs for identifying reliable HKG.

## Materials and Methods

### Surgery

Adult female Wistar rats (weighing approximately 200 g) were divided into three experimental groups: (i) control uninjured animals (n = 4); (ii) animals which underwent median nerve crush injury (axonotmesis, n = 3 for each time-point); (iii) animals which underwent end-to-end repair (neurotmesis, n = 3 for each time-point). For the surgeries, rats were put under deep anesthesia using Tiletamine and Zolazepam (Zoletil) i.m. (3 mg/kg). In the crush group, the crush lesion was applied at the middle of the arm, using a non-serrated clamp, by compressing the nerve for 30 seconds, as previously described [17]. In the end-to-end repair group, the nerve was transected and the proximal and distal nerve stumps were immediately sutured together. For each animal, both left and right median nerves underwent injury/repair.

Rats were sacrificed by lethal i.m injection of Tiletamine and Zolazepam at different time-points after injury/repair (1, 3, 7, 14 days, and 4 weeks for the nerve crush, and 1, 3, 7, 14, days, 4, 8 and 12 weeks for the end-to-end repair). 10 mm-long nerve segment distally to the lesion site was collected.

### Ethics Statement

All procedures were approved by the Bioethics Committee of the University of Torino, by the Institutional Animal Care and Use Committee of the University of Torino, and by the Italian Ministry of Health, in accordance with the European Communities Council Directive of 24 November 1986 (86/609/EEC).

Animals were housed in the animal facility of the Neuroscience Institute Cavalieri Ottolenghi (NICO, Orbassano, University of Torino) in plastic cages with free access to food and water; their room was maintained at constant temperature and humidity under 12–12 h light/dark cycles. Adequate measures were taken to minimize pain and discomfort.

### GeneChip Expression Profiling Data

Expression profiling data obtained from mouse injured sciatic nerve samples were downloaded from Gene Expression Omnibus (GEO) web site (http://www.ncbi.nlm.nih.gov/geo/).

Published microarray data from three independent analyses were found and analyzed:

1. Series Accession # GSE33454, corresponding to control sciatic nerves compared with injured sciatic nerves 1 and 5 days after nerve cut [14], analyzed on Illumina MouseRef-8 v2.0 expression beadchip, platform GPL6885, containing 25697 oligonucleotide probes. Adult female C57BL6/J mice sciatic nerves were transected using surgical scissors. A sham-operated control included the sciatic nerve exposure, without any additional manipulation.
2. Series Accession # GSE386932, corresponding to control nerves compared with injured nerves 7 days after sciatic nerve cut [15], analyzed on GeneChip Mouse Genome 430 2.0 Array (Affymetrix), platform GPL1261, containing 45101 probes. Total RNA was purified from a 10 mm segment of the distal stump and uninjured contra-lateral nerve from control mice 7 days after nerve cut. For each condition, two independent samples (replicates) were generated from pooled nerves of 4/6 mice resulting in a total of 4 samples: CTRL.cut.R1, CTRL.cut.R2, CTRL.uncut.R1, CTRL.uncut.R2.
3. Series Accession # GSE22291, corresponding to control nerves compared with injured nerves 1, 3, 7 and 14 days after sciatic nerve microcrush [16]. Samples were hybridized to GeneChip Mouse Genome 430 2.0 Array (Affymetrix), platform GPL1261, containing 45101 probes. C57BL6 mice underwent a microcrush lesion of their left sciatic nerve. At 0, 3, 7 and 14 days post-injury, sciatic nerves were collected and connective tissue removed; a 4-mm long sciatic nerve segment was taken from the nerve distal stump, starting at 1 mm distal from the lesion up to 5 mm distal. Distal nerve stumps were pooled by group and RNA extracted. Biological replicate was done.

### Analysis of GeneChip Expression Profiling Data

To identify stably expressed genes, GeneChip raw data were downloaded, analyzed and normalized using the Robust Multi-array Average (RMA) approach to obtain the list of analyzed genes, with the corresponding fluorescence at the different time points after nerve lesion (control, 1 day and 5 days; control and 7 days; control, 1, 3, 7 and 14 days), and probe identification, symbol, entrez gene identification, definition, ontology process and function.

Microarrays performed on RNA extracted 1 and 5 days after sciatic nerve cut [14] were firstly analyzed. Fold changes (fc) corresponding to the ratio between (i) fluorescence at 1 day after lesion and fluorescence of control samples (fc_1/0_), (ii) fluorescence at 5 days after lesion and fluorescence of control samples (fc_5/0_), and (iii) fluorescence at 5 days after lesion and fluorescence at 1 day after lesion (fc_5/1_) were calculated. Among them, those with a fc between 0.99 and 1.01 were preserved and the others discarded. To obtain sufficiently expressed genes, all genes with a fluorescence signal <100 were also discarded. Only those genes represented by more than one probe were selected, obtaining a list of candidate genes stable at 1 and 5 days after lesion and repair. Then, microarray performed on RNA extracted 7 days after sciatic nerve cut [15] were analyzed and the ratio between fluorescence at 7 days after lesion and fluorescence of control samples (fc_7/0_) was calculated.

All probes for each selected gene were identified and the average and standard deviation of fc_1/0_, fc_5/0_, fc_5/1_ and fc_7/0_ were calculated; only those genes with a fc average between 0.98 and 1.02 and a standard deviation ≤0.05 were selected.

Finally, microarray performed on RNA extracted 1, 3, 7 and 14 days after sciatic nerve crush [16] were analyzed and the corresponding fc average and standard deviation were calculated to confirm the stability of the previously selected genes.

Primers were designed using the open-source Annhyb software (http://www.bioinformatics.org/annhyb/) and synthesized by Invitrogen (Life Technologies Europe BV, Monza, Italy). Primers sequences are reported in Table 1.

### RNA isolation, cDNA preparation and quantitative real-time PCR

Total RNA was isolated using the TRIzol Reagent (Invitrogen) according to the manufacturer's instructions. Briefly, 0.75 µg total RNA were subjected to a reverse transcriptase (RT) reaction in a 20 µl reaction volume containing: 1× RT-Buffer (Invitrogen), 0.1 µg/µl bovine serum albumin (BSA), 0.5 µM dNTPs; 7.5 µM random decamers (Invitrogen); 40 U RIBOlock (Fermentas) and 200 U SuperScript III Reverse Transcriptase (Invitrogen). The reaction was performed at 25°C 10 min, at 50°C 90 min, at 70°C 15 min. Quantitative real-time PCR was performed using an ABI Prism 7300 (Applied Biosystems, Life Technologies Europe BV, Monza, Italy) detection system. cDNA was diluted 12.5 folds in nuclease-free water and 5 µl (corresponding to 15 ng starting RNA) were analyzed in a 20 µl reaction volume, containing 1× iTaq Universal SYBR Green Supermix (BioRad) and 300 nM forward and reverse primers. Dissociation curves were routinely performed to check for the presence of a single peak corresponding to the required amplicon. Analysis was performed in technical and biological triplicate.

Threshold cycles (CT) were obtained and used to calculate gene stability by NormFinder [1] and geNorm [5] algorithms.

### NormFinder analysis

To analyze the stability of the candidate genes with NormFinder algorithm [1], the CT values must be transformed into relative quantification data using the ΔCT method: for each candidate gene, the ΔCT value must be calculated for all the samples subtracting the highest CT value (corresponding to the sample with the lower gene expression) from all other CT values (the sample with the lower gene expression will thus have ΔCT = 0). Then, applying the equation 2^−ΔCT^ to all the samples, the expression of the analyzed gene will be expressed relatively to the sample with the lower gene expression level, which will be equal to 1. The NormFinder software is freely available as a Microsoft-Excel Add-in. 2^−ΔCT^ data must be organized in the spreadsheet. Following NormFinder instructions, all cells must be selected and the software provides the gene list with the corresponding stability value: the lower the value, the higher the gene stability.

### geNorm analysis

To analyze gene stability with geNorm algorithm, CT values must be transformed into relative quantification data using the ΔCT method as described above for NormFinder. We developed a software to calculate the gene-stability measure M that allows to analyze the raw CT values, that must be organized into a Microsoft Excel or OpenDocument spreadsheet with the sample names in the first column and the gene names in the first row. The software calculates the 2^−ΔCT^ values for all the samples and then, applying the algorithm developed by Vandesompele, provides the list of the most stable genes with the corresponding stability measure M: the lower the M, the higher the gene stability.

According to this algorithm, a candidate gene is compared to each of the other candidate genes. For each sample, the log_2_ of the ratio between the relative expression of the candidate gene and the relative expression of the other candidate genes is calculated. Then, the standard deviation for each set of samples is calculated; the arithmetic mean of all standard deviations corresponds to the gene-stability measure M. This calculation is repeated for each candidate gene. Genes with the lowest M values have the most stable expression. Following exclusion of the gene with the highest M value, the M value is measured again, and again the gene with the highest M value is excluded. These steps are repeated until only two genes with the lowest M value are obtained.

The lower number of HKG required for reliable normalization, that is defined by a normalization factor (NF), was also determined; the NF was firstly calculated as the geometric mean of the expression level of the two most stable genes and then re-determined adding each time the next most stable gene. The pairwise variation, Vn/Vn+1, was calculated between two consecutive NF to determine the effect of the addition of a new gene on the NF. According to geNorm, a V value lower than 0.15 has been recommended as the optimal score. This means that a pairwise variation higher than 0.15 indicates that the addition of a HKG has a significant effect on normalization and should be included for calculation of a reliable NF. Additional reference genes should be included to the NF until the added gene has no significant effect to the newly calculated NF.
